# Supplementary material for: Controversy in the Use of CD38 Antibody for Treatment of Myeloma: Is High CD38 Expression Good or Bad?
Source: Cells. 2020 Feb 6;9(2):378. doi: 10.3390/cells9020378 (PMC7072398; doi:10.3390/cells9020378)
Supplement: Supplementary file 1 [file cells-09-00378-s001.pdf]

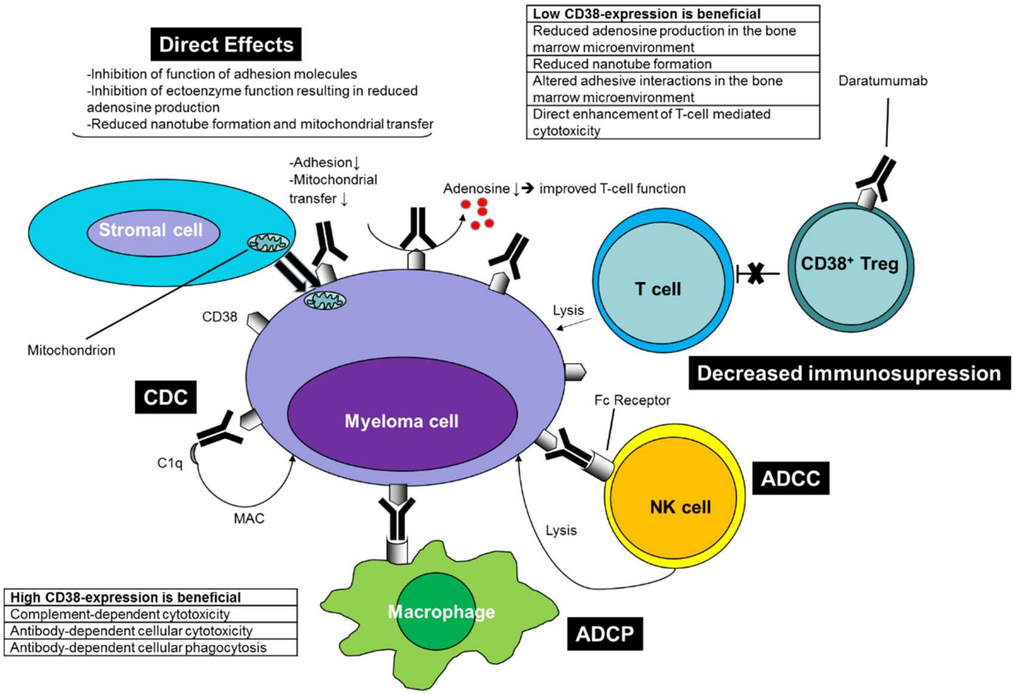

Figure S1. Mechanisms of action of daratumumab. Daratumumab has potent anti-myeloma activity through mechanisms of action that are dependent on high CD38 expression levels on tumor cells (CDC, ADCP and ADCC). Daratumumab also depletes CD38-positive immune suppressor cells such as regulatory T cells, which may result in an improved host-anti-tumor immune response. In addition, daratumumab treatment results in a rapid reduction of CD38 levels on the cell surface of myeloma cells. The daratumumab-mediated CD38 downregulation on myeloma cells may result in several beneficial effects including decreased adenosine production, reduced nanotube formation and mitochondrial transfer, and altered adhesive interactions in the bone marrow microenvironment.

Abbreviations: MAC: membrane attack complex; C1q, complement component 1q; CDC, complement-dependent cytotoxicity; ADCC, antibody-dependent cellular cytotoxicity; ADCP, antibody-dependent cellular phagocytosis; Treg, regulatory T cell.
